# Transitional care in clinical networks for young people with juvenile idiopathic arthritis: current situation and challenges

**DOI:** 10.1007/s10067-015-2950-x

**Published:** 2015-04-30

**Authors:** Mary Cruikshank, Helen E. Foster, Jane Stewart, Joyce E. Davidson, Tim Rapley

**Affiliations:** Institute of Cellular Medicine, The Medical School, Newcastle University, Newcastle upon Tyne, NE2 4HH UK; School of Medical Education, Newcastle University, Newcastle upon Tyne, UK; Scottish Paediatric and Adolescent Rheumatology Network, Glasgow, Scotland UK; Institute of Health and Society, Newcastle University, Newcastle upon Tyne, UK

**Keywords:** Clinical networks, Juvenile idiopathic arthritis, Paediatric and adolescent rheumatology, Qualitative research, Transition

## Abstract

Clinical networks for paediatric and adolescent rheumatology are evolving, and their effect and role in the transition process between paediatric and adult services are unknown. We therefore explored the experiences of those involved to try and understand this further. Health professionals, young people with juvenile idiopathic arthritis and their families were recruited via five national health service paediatric and adolescent rheumatology specialist centres and networks across the UK. Seventy participants took part in focus groups and one-to-one interviews. Data was analysed using coding, memoing and mapping techniques to identify features of transitional services across the sector. Variation and inequities in transitional care exist. Although transition services in networks are evolving, development has lagged behind other areas with network establishment focusing more on access to paediatric rheumatology multidisciplinary teams. Challenges include workforce shortfalls, differences in service priorities, standards and healthcare infrastructures, and managing the legacy of historic encounters. Providing equitable high-quality clinically effective services for transition across the UK has a long way to go. There is a call from within the sector for more protected time, staff and resources to develop transition roles and services, as well as streamlining of local referral pathways between paediatric and adult healthcare services. In addition, there is a need to support professionals in developing their understanding of transitional care in clinical networks, particularly around service design, organisational change and the interpersonal skills required for collaborative working.

*Key messages*

• *Transitional care in clinical networks requires collaborative working and an effective interface with paediatric and adult rheumatology*.

• *Professional centrism and historic encounters may affect collaborative relationships within clinical networks*.

• *Education programmes need to support the development of interpersonal skills and change management, to facilitate professionals in networks delivering transitional care*.

## Introduction

Delivering the ‘right care, at the right time, in the right place, by the right people’ is a challenge for all healthcare services [1], but particularly so for subspecialties, such as paediatric and adolescent rheumatology, where inequities in access to specialist care are known to exist [2]. In the UK, to help address this and to facilitate delivery of specialist care closer to a patient’s home, there is a support for development of paediatric subspecialty clinical networks [1, 3]. Broadly defined, clinical networks are ‘linked groups of health professionals and organisations … working in a coordinated manner, unconstrained by existing and professional and organisational boundaries to ensure equitable provision of high quality clinically effective services’ [4]. This collaborative way of working is now recommended, and there are a number of paediatric and adolescent rheumatology clinical networks evolving across the UK [5].

Transition in terms of health is defined as a young person-centred process and addresses the medical, psychosocial and vocational issues during the move of a young person with a chronic disease from child to adult-focused clinical care [6]. In the UK and other parts of the world, adult rheumatologists have historically provided healthcare for children with rheumatic disease [7]. Along with postgraduate training programme changes and retirement of adult rheumatologists, an increasing number of children are now being managed first and foremost by paediatric trained professionals [8, 9]. As a result, a move will be required to transfer a young person’s care to adult services, to a new healthcare setting, new healthcare providers, or both. It is acknowledged that transition is much more than the event of the transfer of care between paediatric and adult care [10], and for young people with juvenile idiopathic arthritis (JIA), who continue to have active disease [11–13], having support and continuity of specialised healthcare personnel throughout the time of adolescence and young adulthood is important [6, 14]. The provision of consistent, coordinated healthcare may impact on young people’s engagement with healthcare services and biomedical outcomes [15–17]. There is no single gold standard model for transitional care [10, 18–20]. However, there is an emerging evidence-base of important core principles, such as key issues that are central to young people’s positive experience of care [21]. However, transitional care is dependent on local healthcare personnel, infrastructure and funding arrangements, often leaving patients with unmet needs [18, 22]. It is unknown whether or how clinical networks impact on the provision of transitional care and the transfer process. We therefore explored the experiences of those involved to try and understand this further.

## Methods

We report on a component of a wider ongoing study about education in clinical networks for JIA. The wider study employed a range of qualitative methods: focus group discussions and one-to-one interviews. The study had Research Ethics Committee approval (NRES Committee North East—Sunderland; ref. 12/NE/0338) and conformed to NIHR requirements.

### Setting

Potential health professional participants were identified and recruited via five NHS tertiary centres: three in England and two in Scotland, the British Society for Paediatric and Adolescent Rheumatology, and five paediatric and adolescent rheumatology clinical networks/local hospitals with shared care arrangements. Young people with JIA and their families were recruited via two charities: Arthritis Care and the Scottish Network for Arthritis in Children.

### Sample

Seventy participants were recruited and interviewed. Backgrounds included paediatric rheumatologists (*n* = 11), paediatricians (*n* = 10), adult rheumatologists (*n* = 2), adolescent rheumatologist (*n* = 1), paediatric trainees (*n* = 2), paediatric rheumatology nurses (*n* = 9), paediatric rheumatology physiotherapists (*n* = 3), paediatric rheumatology occupational therapists (*n* = 3), young people with JIA—median age 15 years, range 12–17 years (*n* = 8), and parents/carers of children and young people with JIA (*n* = 21). Sampling was designed to ensure variation in professional background, network geography, structure and stage of development. Focus groups were held with young people with JIA and separately with parents/carers of children and young people with JIA who had experience of paediatric rheumatology care from different services across a network.

### Data collection

Seventy participants took part in nine focus groups and ten one-to-one interviews. One participant took part in a focus group as well as an interview. Focus groups and interviews were semi-structured, lasting 30–90 min, and conducted face-to-face by a clinical research fellow, at locations suiting the participants. The focus group facilitator/interviewer was independent of the clinical care of the young people who participated. Focus groups and interviews were digitally recorded, transcribed verbatim and anonymised. Field notes were written, providing additional resource for analysis.

### Data analysis

Transcripts were examined carefully and repeatedly to identify patterns and ideas and involved open and focused coding, constant comparison, deviant case analysis, memoing, and mapping [23]. Data segments were also analysed and discussed with the project team exchanging and testing interpretations.

### Data presentation

Excerpts from transcripts are given, with reference to origin: focus group (FG) or interview (I) and line number. Emphasis from original audio-recording is added in italics. In the interest of confidentiality, exact professional background of the origin of quotes is only disclosed if the background is significant in giving further context to the point being expressed.

## Results

### ‘A big gap in an awful lot of places’: inequities in transitional care

A range of network transitional care descriptions was found, ranging from ‘brilliant’ (FG 7.1; 362) with some centres having ‘very clear transitional pathways’ (I 6.1; 118) to others with ‘not very clear’ (FG 8.1; 631) services, and ‘a big gap in an awful lot of places’ (FG 7.1; 366). One centre previously had ‘a good service’ but this had now ‘collapsed’ (FG 9.1; 469). These descriptions related to healthcare professional’s perception about the holistic transitional care process.

However, the main focus of discussion was in relation to one part of transition—the transfer of care from paediatric to adult care. Echoing findings from previous research [24], provision of designated transfer or hand over clinic in a network were not a universal occurrence. There was a spectrum of involvement from adult rheumatology, ranging from an adult rheumatologist joining the paediatric team to see ‘the last few [teenage] patients at every [network] clinic’ (FG 7.1; 371), to a local paediatrician resorting to ‘writing to the GP [to] refer them to an adult rheumatologist’ (FG 8.1:637). In centres without formalised transfer arrangements, there was acknowledgement by professionals from all backgrounds that joint consultations with adult and paediatric teams coming together were ‘ideally how it should be’ (FG 8.1; 638).

Although some teams had prepared young people to manage their own consultations ‘without [their] parents’ (I 1.1; 565) and had documented transition plans, one team acknowledged that they just ‘put’ patients straight into the adult service, akin to going ‘with nothing’ (ERF FG 9.1; 473).

### ‘It’s on the horizon’: transitional services evolving

We found that clinical networks predominately focused initially on providing (in a variety of different ways) specialist care locally and focus on transitional care and youth friendly services was not an early priority for paediatric rheumatology teams. However, as networks have evolved, transitional care services are ‘beginning to happen’ (FG 7.1; 348).

Restructuring of healthcare provision in some regions has resulted in a shift of children’s care (or planned shift) away from an adult-led rheumatology service to one which is more appropriately ‘paediatrically driven’ (FG 7.1; 190). For these services, there was then a need to develop a more formalised transfer of care arrangement. This had previously not been required as some young people with JIA had ‘never met’ (FG parents 2.1; 273) a paediatric rheumatologist, because ‘they all stay with me [adult rheumatologist)]’ (I 8.1; 433). With reconfiguration of services, establishment of a transition service was ‘on the horizon’ (I 8.1; 442).

### ‘Transition … that works brilliantly’: what makes the transition process work

A number of factors related to the healthcare infrastructure and certain characteristics of the individuals involved were reported to make the transfer part of transition process in a clinical network ‘work brilliantly’ (FG 7.1; 362), or more easily.

#### Healthcare infrastructure

For paediatric rheumatologists who had experience of working across a number of centres, establishment of a transition clinic was easier if one adult rheumatologist per centre acted as the receiving clinician and there was ‘only one hospital to transition to’ (FG 7.1; 398).

#### Individual characteristics

‘Effective transition’ (FG 7.1; 359) was unsurprisingly easier with enthusiastic, motivated, and interested professionals from both paediatric and adult services acting together as advocates for young people. Good interpersonal relationships of key individuals helped form these links between paediatric and adult services, facilitating transfer of care frequently on a ‘patient-by-patient basis’ (I 6.1; 131). Even when there were multiple referral options, the preference was based on prior experience, and knowing ‘who [would] care for [them] in a way that [paediatric teams] want them to’ (FG 7.1; 413). Flexibility of healthcare professionals and ‘individual transition plan[s]’ (FG 7.1; 361) was evident particularly in smaller network clinics, where there were only a few patients requiring transfer; designated fixed transition clinics were recognised as not necessary. In these centres, transfer arrangements were made on an as required individual basis, determined by discussions between paediatric and adult multidisciplinary teams.

### ‘Things that can be tricky’: challenges in the transfer process

A number of challenges or ‘things that can be tricky’ (I 10.1; 156) were encountered in relation to the transfer of care between paediatric and adult services in clinical networks. These included workforce shortfalls, differences in service priorities and healthcare infrastructures, standards of care and historic encounters.

#### Workforce shortfalls

Services were vulnerable around the time of staff retirement when there was a delay in appointing a replacement or when ‘there’s no one to do it because [the paediatrician] didn’t have any hours’ (FG 9.1; 500) for transition in their job plan. This workforce shortfall included staff in both adult and paediatric services and also the MDT; ‘it was both medical and nursing, they couldn’t get anyone in nursing or anyone continuous to do it’ (FG 9.1; 479).

#### Service priority differences

Transition service development was affected when professionals, (from both adult and paediatric teams), and management had other more pressing priorities. ‘We agreed, as a network, what our priorities were’ (I 7.1; 191). Establishing links in ‘areas maybe where a network is a new idea that hasn’t been perceived to be needed in the past’ (I 5.1; 274) has taken time as paediatric rheumatologists ‘enter into negotiations to then try and arrive at that in a way that is acceptable to both parties’ (I 5.1 264).

#### Healthcare infrastructure mismatch

Development of designated transition clinics and transitional roles was difficult if adult healthcare services used young person’s postcodes to dictate where they are referred. ‘Even though there are interested people, they’re only allowed to see the people who go into their geographic bit of the region.’ (FG 7.1: 428). So, locality was prioritised over a young person’s specific needs.

#### Standard differences

Differences between a specialist and local care provision were more noticeable when network models involved professionals physically working between different hospitals. Although ‘closer to home care’ was acknowledged to be better for families, when professionals perceived that their specialist service was better than that provided locally, frustration in not being able to offer the same standard of care was apparent.

‘if they travelled here they would get a better service so it might be convenient for them to be seen locally but I’m not sure that’s the same *level* of service that they would be getting if they came here’ (FG 1.1; 231).

This view may be justified for specialist centres who had accreditation for their transition service (for example, ‘You’re Welcome’ status, a UK Department of Health initiative of Quality Criteria for young people friendly health services [25]). However, the ‘view that theirs [transition service at the specialist centre] is better…. is that’s something that we [the local team] just keep developing and working on’ (I 10.1; 185).

In one specialist centre, around the age of 12–14 years, an early transfer of care occurred from the paediatric centre to an adolescent centre. Up until this time, the paediatric specialist centre shared care with local link general paediatricians providing care closer to home. However, one link paediatrician had found that few, if any, young people’s care was transferred to their local adult rheumatology services. This link paediatrician had experienced that ‘they [local adult rheumatology] really do *not* like shared care; they want one consultant having the ownership and not sharing the care with someone else’ (FG 8.1: 631). Reasons for this included the suggestion that the adult rheumatologist had voiced that they had ‘had exactly the same training’ as clinicians from the specialist centre, and ‘d[idn’t] see kids going back to [the specialist centre] and then coming here locally as different’ (FG 8.1; 692).

#### Personal encounters

Difficulties in establishing smooth transfer arrangements involved ‘much more thorny issues’ (I 6.1; 112) and even conflict, when professionals (from both paediatric and adult rheumatology) had ‘a difficult time’ (I 9.1; 285) during network establishment. This was particularly noticeable when care had been shifted or ‘taken’ (FG 1.1; 398) away from adult to paediatric services. This change in service delivery was welcomed by some adult rheumatologists as ‘we’re not very good at talking, thinking around paediatric side’ (I 8.1; 86), but harder to accept by others, as ‘it was my thing. It was, and I’d been doing it for all those years’ (I 9.1; 286). Despite shifting roles towards the adolescent age group and transition, the establishment of clinical networks had in some areas adversely affected relationships.

One specialist paediatric team

‘had a couple of patients from [a local hospital]…. who were very poorly and came to us for specialist care and you know we are talking a couple of years in … in the care [of a general paediatrician or adult rheumatologist]’ (FG 4.1; 433).

They had found it difficult to ‘send them back to local care’ (FG 4.1; 436). One young person was now nearing the age for her care to be transferred to adult services, and the specialist team had found that they were

‘actually almost having to persuade them that they are going to get good care there [back at their local centre] (FG 4.1; 444).

Transferring back to their original local service was a perceived potential problem, particularly if there were no strong links or confidence in local services. This clearly highlights the need for collaborative working.

## Discussion

Despite universal agreement that good transitional health care is necessary, including recommendations for transitional care for young people with JIA [5], our findings add to the evidence that transition is not always done well and needs to be improved [22, 26]. A variety of transitional programmes exist and it is acknowledged that core principles, rather than a single model, for transitional care services are required [18, 19, 21, 27]. In the context of transition in diabetes care, it has been suggested that continuity of the provision of service elements across paediatric and adults services and the continuity in relationships with professionals, alongside care that is flexible and responsive to individual needs, are central facets to young people’s experience [28]. Where continuity across services is lacking, more work has to be undertaken with young people to enable them to manage the transfer.

Little is known about the effect that clinical networks have on the transition process. As clinical networks evolve, there is expectation that their establishment will help address inequities in access to specialist care, including transition. It is therefore important as clinical networks evolve, that adult care providers are also involved in network service development, so as to better enable continuity of care for young people. Currently, transitional care lags behind other service development areas [21]. The priority of clinical networks has been to improve access to paediatric rheumatology multidisciplinary teams, and our findings emphasise that there are still inequities to be solved for many children and young people with JIA.

The findings from this study raise three specific issues relating to implementation of transitional care in clinical networks: the nature of service design and quality assessment, the effect of organisational change and the practicalities of collaborative working.

Firstly, clinical networks highlight the need for flexibility of service design. Not only does the transition process require tailoring for each individual [29], transfer of care needs to be tailored, without compromising care quality, to what practically works for local services. When care is delivered in clinical networks, patients may be geographically dispersed, resulting in small numbers requiring transition; designated transition clinics may not be needed. Rather, transition should be embedded in the local delivery of high-quality care for all young people; designated transition clinics may not actually be a good benchmark for service comparison and quality assessment [24]. Flexibility may be needed to override referral pathways that do not facilitate healthcare provision appropriate to a child’s development. Although ‘developmentally appropriate health care’ is ill defined [30], provision of consistent, coordinated healthcare throughout adolescence and young adulthood is a key issue which impacts on a young person’s engagement with healthcare services and their biomedical outcomes [15, 16]. Appropriate care for adolescents and young adults is potentially different from paediatric as well as adult care [18–20, 31], and there is an expectation that the healthcare setting is adapted to their needs [32]. A network which is ‘unconstrained by existing and professional and organisational boundaries’ [4] may facilitate this developmentally appropriate approach.

Secondly, there may be differences in acceptance during organisational change caused by differences in perception of service qualities. Care closer to a patient’s home may have benefits, but this may cause tensions if there are evident or perceived differences in transition service standards across the network. The notion that ‘our service is better’ may come from a professional-centric perspective, believing that one’s own professional group, hospital or service is ‘superior’ to another, creating tension between individuals and organisations. Although the concept is more familiar in the context of interprofessional education [33], lessons may be learnt by developing shared understanding of current provision and exploring ways to improve transitional care services together.

Thirdly, there is a requirement for collaborative working to ensure a smooth transfer for the young person. Previous research highlights a need to improve communication between paediatric and adult healthcare providers during transition [34]. Communication difficulties may be exacerbated by the specific issues arising during network establishment surrounding the legacy of historic encounters and shifted roles affecting this relationship. Network establishment and resultant organisational change may for some require a new way of working. Our research suggests the need to help professionals to manage this change and supports previous work identifying desire for interpersonal skill training that facilitates professional working in a clinical network [35].

We acknowledge that this study has its limitations, particularly with regard to the under representation of the perspective from adult rheumatology professionals and primary care. However, this reflects wider limitations of this type of research, including issues that not everyone who was invited to participate responded to the invitation. However, it is hoped that the findings of this work will still resonate and open discussions with others involved in service development and delivery of adolescent healthcare. Not only do education programmes need to address the skills to work with young people [36], as well as the skills to support young people through the transition process, there is a need to support professionals in developing a wider understanding of transitional care in clinical networks, particularly around service design, organisational change and interpersonal skills required for collaborative working.
